# Decolonizing climate agreements strengthens policy and research for all future generations

**DOI:** 10.1038/s41467-024-49143-x

**Published:** 2024-06-06

**Authors:** Graeme Reed, Angele Alook, Deborah McGregor

**Affiliations:** 1 Centre for Indigenous Knowledges and Languages, York University, 4700 Keele Street, Toronto, ON M3J 1P3 Canada; 2 School of Gender Sexuality and Women’s Studies, York University, 4700 Keele Street, Toronto, ON M3J 1P3 Canada; 3 Osgoode Hall Law School, York University, 4700 Keele Street, Toronto, ON M3J 1P3 Canada

**Keywords:** Governance, Social sciences

## Abstract

Global climate policy has increasingly acknowledged the specific contributions of Indigenous Peoples. The outcome of COP 28, however, demonstrates that this acknowledgement has not shifted the conceptual foundations of dominant climate solutions, nor has it created space for Indigenous Peoples to effectively contribute. Drawing on our expertise as Indigenous scholars and practitioners, we offer four recommendations to shift climate policy and research away from these foundations towards reciprocal relationships with the natural world – strengthening it for future generations.

As the frequency and severity of extreme climate events increase across the globe, so-to-does the recognition of Indigenous Peoples, and their knowledge systems, in proposing climate solutions^1^. Within the UN Framework Convention on Climate Change (UNFCCC), references to Indigenous Peoples, self-determination, rights, and traditional knowledge have been growing in decisions texts^2^ and in Nationally Determined Contributions (NDCs)^3^. This trend continued at the twenty-eighth Conference of the Parties (COP 28), where Indigenous Peoples worked closely with the United Arab Emirates (UAE) Presidency to amplify their voice, co-hosting an Indigenous Peoples Dialogue, and supporting an Indigenous Peoples Pavilion. In substantive terms, COP 28 concluded negotiations on several important topics, including the Global Stock Take (GST), the Global Goal on Adaptation, and the Loss and Damage Finance Facility. All decisions can be found here and additional information about the negotiations can be found here.

In each of these negotiations, we saw consistent positions from Indigenous Peoples: full respect for our rights in line with the UN Declaration on the Rights of Indigenous Peoples; the ethical and equitable inclusion of our knowledge systems; our full and effective participation; and concrete action to prevent warming over 1.5 °C. Underlying these positions is a distinct diagnosis that recognizes climate change is a symptom of a deeper problem, driven by the structural legacy of colonialism and capitalism, a shift in human values, and the cumulative impacts from industrialization^4^, contributing to the symptom of runaway emissions. To unpack these distinctions, we discuss the GST decision text–the outcome of a multi-staged process for Parties to reflect on whether (or not) they were taking sufficient action to achieve the goals of the Paris Agreement—as an articulation the dominant approach to climate policy. We then demonstrate how Indigenous Peoples are elevated to positions of importance, while, on the other hand, remain structurally excluded from impacting climate policy. Lack of meaningful Indigenous engagement within these political, financial, epistemological, and relational spaces results in tokenism rather than decolonization. We close with four recommendations to shift climate policy and research away from these technocratic foundations towards rebalancing reciprocal relationships with the natural world^5^.

## Situating the GST within the work of the Paris Agreement

The Paris Agreement, an agreement negotiated in 2015 under auspices of the UNFCCC, commits to hold global average temperature rise to well below 2 °C and pursue efforts to limit the temperature increase to 1.5 °C. There are six references to Indigenous Peoples in the Agreement, including a call for Parties to respect their obligations to human rights and the rights of Indigenous Peoples, and the creation of a platform for the exchange of best practices, now called the Local Communities and Indigenous Peoples Platform. To demonstrate progress, Parties voluntarily submit Nationally Determined Contributions (NDCs), documents that outline their emission reduction targets and their proposed plan to achieve them. Every five years, Parties participate in a stocktaking exercise, called the GST to review their collective progress and determine whether additional steps are required to achieve the identified temperature goal. 2023 was the conclusion of the first GST, clouded by the findings of the annual updated UNFCCC NDC Synthesis Report which confirmed that current pledges will increase emissions by 8.6% by 2030, rather than decrease them. Urgent and transformative climate action, including adaptation, is drastically needed.

Amidst this backstop, and growing calls for rapid emission reductions, Parties concluded the GST process, reflecting on eighteen months of technical work. During the technical and political phase, Indigenous Peoples had limited ability to contribute, participating alongside members of civil society with the ability to make submissions and observe the negotiations. The decision text was heralded by the COP 28 Presidency as “unprecedented” for calling for a “[transition] away from fossil fuels in energy systems, in a just, orderly and equitable manner.” It featured sections on context and cross-cutting concerns; an exploration of progress to meet the temperature goals in areas of mitigation, adaptation, Loss and Damage, and means of implementation (finance); and guidance for the way forward. The path forward, unfortunately, gave no concrete recommendations, nor guidance for Parties to update their NDCs absent procedural requests inviting them to consider preparing additional NDCs with the “greatest ambition”, and to participate in additional meetings on the “Road map to Mission 1.5”. Another GST begins in 2026, with an expected conclusion in 2028.

The GST contains nine textual references to Indigenous Peoples (see Table 1), including inviting Parties to engage Indigenous Peoples in developing climate policy. While constructive, we observe that this model of tweaking existing approaches to climate policy deepens the reliance on “…technological solutions and market-based approaches that presume a continuation of the structurally inequitable and racist system that led us into this compounding environmental crisis in the first place.” (p. 24)^6^. To avoid this continuation, we examine the GST from four angles–political, financial, epistemological, and relational–to propose four recommendations to advance decolonial climate policy and research.

**Table 1:** Textual References to Indigenous Peoples in the Global Stocktake Decision

Paragraph	Textual References to Indigenous Peoples
Preamble	*Acknowledging* that climate change is a common concern of humankind and that Parties should, when taking action to address climate change, respect, promote and consider their respective obligations on human rights, the right to a clean, healthy and sustainable environment, the right to health, “**the rights of Indigenous Peoples”**, local communities, migrants, children, persons with disabilities and people in vulnerable situations and the right to development, as well as gender equality, empowerment of women and intergenerational equity.
9.	*Reaffirms* that sustainable and just solutions to the climate crisis must be founded on meaningful and effective social dialog and participation of all stakeholders, including “**Indigenous Peoples”**, local communities and governments, women, and youth and children, and notes that the global transition to low emissions and climate-resilient development provides opportunities and challenges for sustainable development and poverty eradication;
55	*Encourages* the implementation of integrated, multi-sectoral solutions, such as land-use management, sustainable agriculture, resilient food systems, nature-based solutions and ecosystem-based approaches, and protecting, conserving and restoring nature and ecosystems, including forests, mountains and other terrestrial and marine and coastal ecosystems, which may offer economic, social and environmental benefits such as improved resilience and well-being, and that adaptation can contribute to mitigating impacts and losses, as part of a country-driven gender-responsive and participatory approach, building on the best available science as well as “**Indigenous Peoples’ knowledge”** and local knowledge systems
61	*Stresses* the importance of global solidarity in undertaking adaptation efforts, including long-term transformational and incremental adaptation, towards reducing vulnerability and enhancing adaptive capacity and resilience, as well as the collective well-being of all people, the protection of livelihoods and economies, and the preservation and regeneration of nature, for current and future generations, in the context of the temperature goal referred to in Article 2 of the Paris Agreement, and that such efforts should be inclusive in terms of adaptation approaches and taking into account the best available science and “**the worldviews and values of Indigenous Peoples”**, to support achievement of the global goal on adaptation;
63 (g)	*Protecting* cultural heritage from the impacts of climate-related risks by developing adaptive strategies for preserving cultural practices and heritage sites and by designing climate-resilient infrastructure, “**guided by traditional knowledge, Indigenous Peoples’ knowledge”** and local knowledge systems;
116	*Recognizes* the role of “**the Local Communities and Indigenous Peoples Platform** in **strengthening the capacity of Indigenous Peoples”** and local communities to effectively engage in the intergovernmental process under the Paris Agreement and calls on Parties “**to meaningfully engage Indigenous Peoples”** and local communities in their climate policies and action;
158	*Acknowledges* the important role and active engagement of non-Party stakeholders, particularly civil society, business, financial institutions, cities and subnational authorities, “**Indigenous Peoples”**, local communities, youth and research institutions, in supporting Parties and contributing to the significant collective progress towards the Paris Agreement temperature goal and in addressing and responding to climate change and enhancing ambition, including progress through other relevant intergovernmental processes;

## Recommendations for decolonial climate policy and research for all future generations

As one of nine “stakeholder” constituencies, the relegation of Indigenous Peoples to the status of non-party stakeholders allows Parties to control how, and where, Indigenous Peoples are referenced in all COP decisions. In the GST decision, for example, the rights of Indigenous Peoples are only acknowledged in a preambular paragraph, not the operative text on proposed solutions. We are concerned that this oversight enables those proposed solutions to proceed absent rights safeguards; for instance, the proposal of “tripling renewable energy capacity globally” does not include anything to ensure that these practices do not reproduce a form of energy development that dispossessed Indigenous People from the natural world. To avoid this form of ‘green’ colonialism, the UNFCCC must explicitly acknowledge Indigenous Peoples as rights-holders, including, the right to self-determination and free, prior, and informed consent (FPIC), in all decisions and all proposed climate solutions. As policymakers and scholars consider the implementation of the solutions proposed in the GST, we encourage their scholarship, and the support for these technologies and markets, is reviewed through a rights-based lens to uphold Indigenous self-determination and participation in decision-making.

Indigenous Peoples face significant constraints in the UNFCCC process, including a lack of access to badges, interpretation, and funding to attend meetings^7^. These barriers, coupled with the delegitimization of Indigenous knowledge systems, prevent meaningful participation in negotiations and meetings. As a result, decisions, such as the operationalization of the Loss and Damage Financial Facility (“Finance Facility”), proceed without our valuable input. Loss and Damage, within the corridors of the UNFCCC, is designed as a fiscal relationship between Annex 1 and Annex 2 Countries (as described in the UNFCCC), which structurally excludes those Indigenous Peoples from Annex 1 Countries (i.e., Arctic and Pacific). As Parties shift their mind to implementation of the Finance Facility, and to the negotiation of the New Collective Quantified Goal, we call for direct funds, including the design of new direct-access mechanisms, to all Indigenous Peoples irrespective of their region.

The GST is replete with climate solutions, such as carbon capture, utilization, and storage, and zero-emission vehicles, designed to address the problem of climate change as a one-dimensional reduction of emissions. By contrast, Indigenous Peoples define climate change as a symptom of a deeper problem, requiring a reset between humans and their relationship with the natural world^5,8^. This ontological disjuncture often results in the marginalization of Indigenous Peoples, and their knowledge systems, within discussions of climate solutions. A paradigm shift is required, or we will continue to be and forced to fit within an ontological path that is destroying the natural world sidelined^9^. Scholars and policymakers must then work to build respectful frameworks of knowledge co-production, in advance of the next GST to inform the technical and political phases. This will uphold the work of Indigenous Peoples to revitalize Indigenous lifeways and Indigenous economies, while also providing tangible contributions to the process’ ability to engage with diverse knowledge systems.

We close with a quote from Great-Grand Mother Mary Lyons, shared at the Annual Indigenous Knowledge Holders Gathering at COP 28, who challenged us to think about our relationship with the natural world: *We must be the good caretakers, and not the bad landlords. It’s not just Indigenous Peoples, it’s all human beings. It’s all plant life, it’s all our water relatives, our sky relatives. We are all related*. In this framing, humans are a part of, not separate from, the natural world. To return to this reciprocal relationship, we must confront the systems of power and domination that continue to legitimize the dispossession of Indigenous Peoples (and other structurally oppressed groups) from the Land^10^. We call on policymakers and scholars to embed a decolonial lens within discussions at the UNFCCC, including preparations for the next GST, as well as their policy and research programmes, to unpack the impact of the structural legacy of colonialism and capitalism on any proposed solution.

## Looking towards the future

Indigenous Peoples engagement in UNFCCC is not just about inclusion in non-Indigenous processes. We have tangible, transformative and innovative proposals, grounded in our knowledge systems and reciprocal relationship with the natural world, that actively strengthens climate policy and research by challenging the dominant assumptions underpinning mainstream climate policy solutions, including those discussed at the UNFCCC. Through an analysis of the GST, we call on policymakers and scholars to shift their thinking to promote reciprocal relationships with the natural world. This is the only way, collectively, we can create a transformative, decolonial, and just future for the next seven generations.
